# Development and validation of an optimized marker set for genomic selection in southern U.S. rice breeding programs

**DOI:** 10.1002/tpg2.20219

**Published:** 2022-05-25

**Authors:** Tommaso Cerioli, Christopher O. Hernandez, Brijesh Angira, Susan R. McCouch, Kelly R. Robbins, Adam N. Famoso

**Affiliations:** ^1^ H. Rouse Caffey Rice Research Station Louisiana State Univ. Agricultural Center Rayne LA 70578 USA; ^2^ Section of Plant Breeding and Genetics, School of Integrative Plant Sciences Cornell Univ. Ithaca NY 14850 USA; ^3^ Cornell Institute for Digital Agriculture Cornell Univ. Ithaca NY 14850 USA

## Abstract

The potential of genomic selection (GS) to increase the efficiency of breeding programs has been clearly demonstrated; however, the implementation of GS in rice (*Oryza sativa* L.) breeding programs has been limited. In recent years, efforts have begun to work toward implementing GS into the Louisiana State University (LSU) Agricultural Center rice breeding program. One of the first steps for successful GS implementation is to establish a suitable marker set for the target germplasm and a reliable, cost‐effective genotyping platform capable of providing informative marker data with an adequate turnaround time. The objective of this study was to develop a marker set for routine GS and demonstrate its effectiveness in southern U.S. rice germplasm. The utility of the resulting marker set, the LSU500, for GS applications was demonstrated using four years of breeding data across 7,607 experimental lines and four elite biparental populations. The predictive ability of GS ranged from 0.13 to 0.78 for key traits across different market classes and yield trials. Comparisons between phenotypic selection and GS within biparental populations demonstrates similar performance of GS compared with phenotypic selection in predicting future performance. The prediction accuracies obtained with the LSU500 marker set demonstrates the utility of this marker set for cost‐effective GS applications in southern U.S. rice breeding programs. The LSU500 marker set has been established through the genotyping service provider Agriplex Genomics, and in the future, it will undergo improvements to reduce the cost and increase the accuracy of GS.

**Abbreviations**
7K: C7AIR rice SNP array
AmpSeq: amplicon sequencing
BLUE: best linear unbiased estimate
CV: cross‐validation
DP: diversity panel
GBS: genotyping‐by‐sequencing
GEBV: genomic estimated breeding value
GS: genomic selection
HRCRRS: H. Rouse Caffey Rice Research Station
LD: linkage disequilibrium
LSU: Louisiana State University
MAF: minor allele frequency
MAS: marker‐assisted selection
PYT: preliminary yield trial
RGS: representative germplasm set
SNP: single‐nucleotide polymorphism

## 1 INTRODUCTION

DNA marker information is widely used for implementing marker‐assisted selection (MAS) to improve the efficiency and precision of conventional plant breeding programs in different crops including rice (*Oryza sativa* L.) (Collard & Mackill, 2008). The utility of MAS is greatest when the target of selection involves a single gene or locus of large phenotypic effect and is often used to introgress useful traits into breeding populations (Cobb et al., 2019). However, many important traits in a plant breeding program, including yield and quality, are inherited in a highly quantitative manner and are determined by many genes of small effect. Thus, MAS is not an effective method for selection of these traits. Highly polygenic traits are often difficult to select because they are affected by the environment and have lower heritability making phenotypic selection in the early breeding stages inefficient.

The introduction of genomic selection (GS), which uses genome‐wide marker information to predict breeding values, has enabled prediction of quantitative traits within breeding populations (Bernardo, 1994; Meuwissen et al., 2001). Genomic selection leverages information from a training population that is both genotyped and phenotyped to train a statistical model that predicts the performance of related individuals referred to as the prediction set. The prediction set is genotyped but not phenotyped, and the genomic estimated breeding value (GEBV) is calculated using genotypic information. The implementation of GS can increase the genetic gain of a breeding program by improving the accuracy of selection, increasing selection intensity, reducing breeding cycle time, and also decreasing the cost of the breeding program operations (Crossa et al., 2017; Heffner et al., 2010; Hickey et al., 2017; Voss‐Fels et al., 2019).

Despite the demonstrated potential of GS to improve the efficiency of rice breeding programs (Grenier et al., 2015; Monteverde et al., 2018; Spindel et al., 2015; Xu et al., 2021), the implementation of GS in applied breeding programs has been limited. This can be attributed to changes in required skill sets and processes within the breeding program that are necessary for routine applications of GS. Among these are the requirement to adopt a data management system for storing and accessing phenotypic and genotypic data, routine use of data analysis and breeding decision‐support tools, optimization of the breeding pipeline, and employment of people with new skill sets within the traditional breeding program (Santantonio et al., 2020).

One of the first steps for successful GS implementation is to establish a suitable marker set for the target germplasm and a reliable, cost‐effective genotyping platform capable of providing informative marker data with fast turnaround time and low cost. The optimal marker density for GS depends on the extent of linkage disequilibrium (LD) across the genome in the target germplasm (Heffner et al., 2009), the number of independent chromosomal segments that need to be tracked in the target population (Daetwyler et al., 2010), and the heritability of the trait (Goddard & Hayes, 2007). In an applied or commercial plant breeding program, the effective population size is often small, leading to strong genome‐wide LD (Flint‐Garcia et al., 2003); therefore, a relatively small number of selected markers is capable of tagging each independent LD block and can be used to predict breeding values with good accuracy. This has been demonstrated in wheat (*Triticum aestivum* L.) (Juliana et al., 2019), barley (*Hordeum vulgare* L.) (Abed et al., 2018), and oilseed rape (*Brassica napus* L. subsp. *napus*) (Werner et al., 2018), where a few hundred to a few thousand markers enable high GS prediction accuracies that are comparable to high marker densities. In addition, breeding programs often breed for different market classes or heterotic groups, creating distinct population structures within the breeding population. Taking advantage of this structure in defining the training populations helps to maintain high LD within the segments and consequently reduce the number of markers required for GS.

Rice is a model crop species, and numerous marker sets are available with varying marker densities; examples include the 1k‐RiCA (Arbelaez et al., 2019), the C7AIR (Morales et al., 2020), the 44K‐single‐nucleotide polymorphism (SNP) chip (Zhao et al., 2011), and the 700K‐SNP High‐Density Rice Array (McCouch et al., 2016). While these arrays represent a rich resource of validated SNPs for genome‐wide association analysis and genetic diversity analysis, they are not ideal for breeding applications where the genetic diversity of the material is relatively narrow. The cost per sample and inflexibility of these arrays hinders their use for routine genotyping for GS where the majority of SNPs tend to be monomorphic in the breeding germplasm. However, these established marker sets represent invaluable resources for identifying tailored subsets of reliable, high‐quality SNPs known to segregate in the target breeding germplasm.

**Core Ideas**

- A SNP marker set was developed for genomic selection in southern U.S. rice breeding programs.
- Predictive ability across target germplasm was shown with 3 yr of data (4,078 lines).
- Within‐population predictive ability was shown across four biparental populations.
- Genomic and phenotypic selection ability to predict future performance was compared.

Sequence‐based genotyping approaches, including genotyping‐by‐sequencing (GBS), have also been used for breeding applications (Poland & Rife, 2012); however, their application requires bioinformatic skills to process the raw data and use the genotypic information, adding an additional layer of complexity for a breeding program.

In recent years, the use of highly multiplexed amplicon sequencing (AmpSeq) technologies (Yang et al., 2016) has provided the opportunity to efficiently genotype hundreds to thousands of selected SNPs at a relatively low cost per sample and offer flexibility in marker set design. Thus, these technologies strike the ideal balance between cost per sample, marker density, and flexibility, making it possible to continually update and optimize the design of the market set. The strategy of designing highly informative, lower density chips for breeding applications using the AmpSeq technology has been recently pursued for global rice, with emphasis on *indica* germplasm (Arbelaez et al., 2019).

Another strategy to reduce the cost while increasing the efficiency of genotyping is to outsource to a genotyping service provider, which eliminates the need to buy and maintain sophisticated equipment and to hire trained personnel for the breeding program. The large‐scale operations of genotyping service providers allow them to reduce the genotyping costs and offer lower prices per sample to consumers. Agriplex Genomics, Diversity Array Technology (DArT), Intertek AgriTech, NRGene Technologies, Eurofins Genomics, and LGC Ltd are examples of such service providers.

Existing rice genotyping marker sets have been previously developed to represent the range of genetic variation within the species and are reliable sources of validated SNPs. We targeted the C7AIR rice SNP array (7K) (Morales et al., 2020) as the primary source of informative and validated SNPs for inclusion in our AmpSeq genotyping platform. The 7K array was developed to detect genome‐wide polymorphism within and between the subpopulations of (Asian) rice, African rice (*O. glaberrima* Steud.), red rice (*O*. *rufipogon* Griff.), and O. *nivara* S. D. Sharma & Shastry. Its ability to detect polymorphism in different species and subpopulations of rice was demonstrated based on genotyping of a set of 544 diverse *Oryza* accessions. Given that our breeding program is focused almost entirely on *tropical japonica* cultivars, we were interested to observe that 2,086 SNPs well distributed across the rice genome were polymorphic in the 74 *tropical japonica* cultivars tested (Morales et al., 2020). Thus, the 7K array was considered sufficient to capture the range of genetic diversity relevant to our breeding program and was used to develop an optimized set of markers for an AmpSeq marker set. An initial set of 1,200 informative and well‐distributed SNPs from the 7K array were identified for the target germplasm, and the LSU1200 marker set was developed for Agriplex Genomics, Cleveland, OH, USA (https://agriplexgenomics.com/) AmpSeq genotyping. Subsequently, a subset of 550 SNPs (LSU500) was selected and validated for GS implementation within southern U.S. rice breeding germplasm.

## 2 MATERIALS AND METHODS

### 2.1 Genetic material and genotyping

A set of 342 lines, the representative germplasm set (RGS), was genotyped with the 7K array (Morales et al., 2020) at the Louisiana State University (LSU) Health Sciences Center in New Orleans, LA. The RGS was selected to represent the genetic diversity of a southern U.S. rice breeding program and consists of 66 modern and historical cultivars, advanced breeding lines, and 276 experimental lines from the largest segment of the target breeding program.

The LSU1200 marker set was developed from a subset of the 7K array and from existing Kompetitive allele‐specific polymerase chain reaction (KASP) assays routinely used in the LSU marker lab. A total of 3,651 lines were genotyped with the LSU1200 marker set through PlexSeq, a mid‐density multiplex next‐generation AmpSeq through the genotyping service provider Agriplex Genomics in Cleveland, OH (https://agriplexgenomics.com/). These lines consisted of 2,067 experimental lines from preliminary yield trials (PYTs) grown at the H. Rouse Caffey Rice Research Station (HRCRRS) in Rayne, LA, from 2017 to 2019, 1,200 lines from four different biparental mapping populations, and 384 lines from a diversity panel (DP) that represents the genetic diversity of the U.S. germplasm, including accessions from different regions of the world (Addison et al., 2021, 2020; Angira et al., 2019). The biparental populations—MPA, MPB, MPC, MPD—originated from eight cultivars representing the elite southern U.S. rice germplasm. These populations were developed through single seed descent in the greenhouse from 2017 to 2019, grown as F_3:4_ panicle rows in 2019, and tested as F_3:5_ plots in 2020 at HRCRRS. The MPA population originated from the cross CL111/‘RoyJ’, MPB from CL153/‘LaKast’, MPC from CL172/‘Cypress’, and MPD from ‘Presidio’/‘Catahoula’. Population MPA consisted in 297 unique row entries and 130 plot entries, MPB in 296 rows and 100 plots, MPC in 279 rows and 100 plots, and MPD in 286 rows and 100 plots.

Finally, 6,406 experimental lines tested from 2018 to 2020 in PYTs and multienvironmental trials were genotyped with the LSU500 marker set through Agriplex Genomics. A summary of the material used in the study is provided in Supplemental Table S1.

### 2.2 Phenotypic data

All PYTs were grown in randomized complete block designs at the HRCRRS, near Crowley, LA (30°14′30″ N, 92°20′46″ W) and were separated according to different grain classes (long grain and medium grain) and different herbicide resistances (Clearfield, Provisia, and conventional). Two rows for each biparental line were grown as F_3:4_ at the HRCRRS in 2019. The 2020 biparental trial was tested with an augmented complete block design at the HRCRRS as F_3:5_ plots. All PYTs and biparental plots were drill seeded in plots 1.42 m wide and 4.12 m long with a heavy‐duty grain drill (ALMACO) at a seeding rate of 100 kg ha^−1^ at a depth of 2 cm. The water management and fertilization followed the standard practices for Louisiana rice (Saichuk, 2014). Grain yield was determined by harvesting the entire plot with a Delta Plot combine (Wintersteiger AG), and a Harvest Master Grain Gauge (Juniper Systems) was used to collect grain weight and moisture. Moisture values of each plot were adjusted to 120 g kg^−1^ water content. Milling samples (100 g) milled on a PAZ laboratory mill (ZaccariaUSA) were used to measure milling yield expressed as grams per kilogram of whole milled kernels over rough rice seed. Days to heading was measured as the number of days between the date of emergence and the day when 50% of the plot or row had panicles emerging from the sheath of the flag leaf. Plant height was measured as centimeters from the soil surface to the panicle tip at maturity; two measures per line were averaged on the rows, and only one measure was recorded for the plots. Chalk, reported as percentage of chalky area of the milled grain, and grain length was measured with SeedCount (Next Instruments). Grain quality traits were measured only on 147 lines for MPA, 112 for MPB, 133 for MPC, and 155 for MPD. Grain yield and milling yield data were not collected on the biparental rows.

### 2.3 Filtering and marker set design

#### 2.3.1 LSU1200 design

Data from the RGS genotyped with 7K array were used to develop the LSU1200 marker set and then further filtered to obtain the LSU500 set. The RGS was split into two sets prior to filtering: key modern and historical lines and the most recent experimental lines from a PYT representing the program's largest market class segment. As the PYT lines made up the majority of the RGS, the data were split and independently filtered to avoid overprioritizing markers that happened to capture the diversity in one year's PYT but may not be as informative for southern U.S. germplasm as a whole. After splitting the data, TASSEL v5 (Bradbury et al., 2007) was used to filter markers for a minor allele frequency (MAF) >0.1 and <20% missing data. After filtering, the –blocks method from PLINK 1.9 (Purcell et al., 2007) was used to identify haplotype blocks with default settings. The haplotype blocks represent groups of SNPs with alleles that are coinherited because of linkage. Haplotype alleles were identified for each block and a custom Python script was used to select the minimum number of SNP markers needed to uniquely identify each haplotype block using a greedy algorithm (Supplemental Code). The ∼1,000 markers chosen from this analysis were combined with trait and genome‐wide markers to obtain the set of 1,218 markers referred to as the LSU1200 (Supplemental Table S2). Pedigree strings from lines grown from 2017 to 2019 were used to identify a set of lines that were representative of the program's diversity. In addition to these lines, lines from several biparental mapping populations and the DP were included to give a total of 3,651 experimental lines. These experimental lines were used to validate the LSU1200 set.

#### 2.3.2 LSU500 design

The LSU500 set was developed by filtering the LSU1200 within the validation set. After removing markers with missing values >10%, the MAF was calculated for all markers. Adjacent markers were considered in pairs, and the markers with the lowest MAF were dropped in each pair to obtain the LSU500 (Supplemental Table S2).

### 2.4 Analysis

All analysis was conducted using the statistical computing software environment R (R Core Team, 2020). Marker density plots were designed with the R package CMplot (Yin et al., 2021). The linear mixed models were fit with ASReml‐R v4 (Butler et al., 2018). The genomic relationship matrix (**K**) was calculated from molecular markers with ASRgenomics (Gezan et al., 2021) using the VanRaden (2008) equation. Outliers in PYTs were removed with studentized residuals above 3 and below −3. The marker subsets were calculated by removing adjacent markers by physical distance. The pedigree‐based additive relationship matrix **A** was obtained with the R package AGHmatrix (Amadeu et al., 2016). The narrow‐sense heritability (*h* ^2^) was calculated as the ratio of additive genetic variance component over the sum of the additive genetic and residual error components as Equation 1:

(1) $$h^{2}=\frac{\sigma_{a}^{2}}{\sigma_{a}^{2}+\sigma_{e}^{2}}$$

$\sigma_{a}^{2}$ is the additive genetic variation calculated fitting **K** in the model, and $\sigma_{e}^{2}$ is the residual error. The broad‐sense heritability (*H* ^2^) was calculated as the ratio of total genetic variance component over the sum of the genetic and residual error components as Equation 2:

(2) $$H^{2}=\frac{\sigma_{g}^{2}}{\sigma_{g}^{2}+\sigma_{e}^{2}}$$

The best linear unbiased predictors or GEBVs were calculated for each single location trial using a single‐trait genomic best linear unbiased predictor with the form Equation 3:

(3) y=Xb+Zu+e

Where *y* is the phenotypic observations, **X** is the incidence matrix of fixed effects, *b* is the intercept, **Z** is the incidence matrix of the random effects, *u* is the random effects, and *e* is the error. It is assumed that $u∼N(0,\sigma_{a}^{2}K)$ and e∼N(0,R), where **K** is the genomic relationship matrix and $R=\sigma_{e}^{2}AR1_{row}⊗AR1_{col}$, where $\mathrm{AR}1_{row}⊗\mathrm{AR}1_{col}$ is the spatial adjustment. For trials with blocks, the block effect *b* was fit as random, with $b∼N(0,\sigma_{b}^{2})$. For every cross‐validation (CV) iteration, 80% of the lines within a trial were selected randomly as the training set, while the remaining 20% were used as the validation set. The same training and validation sets were used for every trait and marker subset iteration. For every trial and trait, the values of 20 CV iterations are presented. The predictive ability is calculated as the Pearson correlation between GEBVs and best linear unbiased estimates (BLUEs); BLUEs were computed with lines as a fixed effect and calculated for each trial individually. These values were calculated using the same model for the GEBVs (Equation 3), but allowing *u* to be fixed instead of random, with all observed records included. The GEBVs from the biparental rows were calculated using a leave‐one‐out CV scheme, where each line was masked and predicted by the remaining lines. No spatial model was used with the rows. The predictive ability of the phenotype was calculated as the Pearson correlation of the 2019 phenotype with the 2020 BLUEs.

The LD was calculated as the squared pairwise correlation (*r* ^2^) of all markers expressed as −1, 0, and 1 of the dosage matrix notations, within each chromosome, and then averaged across a 100‐kb window. The LD was calculated for the PYTs lines and biparental populations separately. Heterozygous markers were set as missing data.

## 3 RESULTS

### 3.1 Marker set design

#### 3.1.1 C7AIR rice SNP array genotyping

To develop a marker set optimized for implementing GS in the southern U.S. rice germplasm, a set of 342 lines, the RGS representing the genetic diversity of a southern U.S. rice breeding program, was genotyped with the rice 7K array (Morales et al., 2020). The RGS consisted of 66 modern and historical cultivars and advanced breeding lines as well as 276 experimental lines from the 2017 Clearfield long‐grain PYT, the largest segment of the breeding program. The 7K array consisted of 7,098 markers, of which 27 failed and 379 had >20% missing data across the RGS. A total of 6,831 markers were polymorphic, with median MAF of 0.03. Sixty percent of the polymorphic markers had a MAF <0.05, 11% between 0.05 and 0.1, 14% between 0.1 and 0.25, 10% between 0.25 and 0.4, and 4% between 0.4 and 0.5 (Supplemental Figure S1). The average distance between the polymorphic markers was 54.3 kb.

The 7K array genotypic data was used to calculate the additive relationship coefficients of the 66 modern and historical cultivars within the RGS lines to correlate them with the coefficients calculated with pedigree information alone. A correlation of 0.67 was observed, providing a baseline for how well higher density marker data can capture relationships compared with pedigree (Supplemental Table S3). The marker data is more effective at capturing the variation present, as it can capture both within‐family and across‐family variation; whereas, the pedigree information is limited to across‐family variation, and pedigree records are not available for some lines.

**FIGURE 1:**
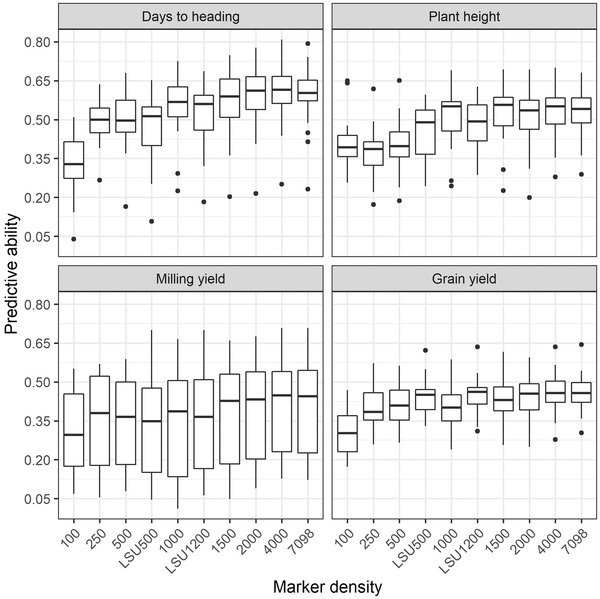
Cross‐validation conducted on the 2017 Clearfield long‐grain preliminary yield trial across different marker densities. Boxplots show the predictive ability for each trait and marker density

A CV to measure the predictive ability of GS across different marker densities, obtained by removing adjacent markers by increasing physical distance, was performed with the 276 experimental lines of the 2017 Clearfield long‐grain PYT to estimate a potential size for the marker set (Figure 1). No major differences in predictive ability (mean and variance) were observed between 7,098; 4,000; and 2,000 markers across all traits. The lack of difference between the 7,098 and 4,000 markers was expected since only 40% of the 7K SNPs had a MAF > 0.05. Milling yield predictive ability started to decrease slightly with 1,000 markers, grain yield with 1,500 markers, days to heading with 1,500 markers, and plant height with 500 markers. The CV variance did not show clear trends across different marker densities.

#### 3.1.2 LSU1200 marker set design

**FIGURE 2:**
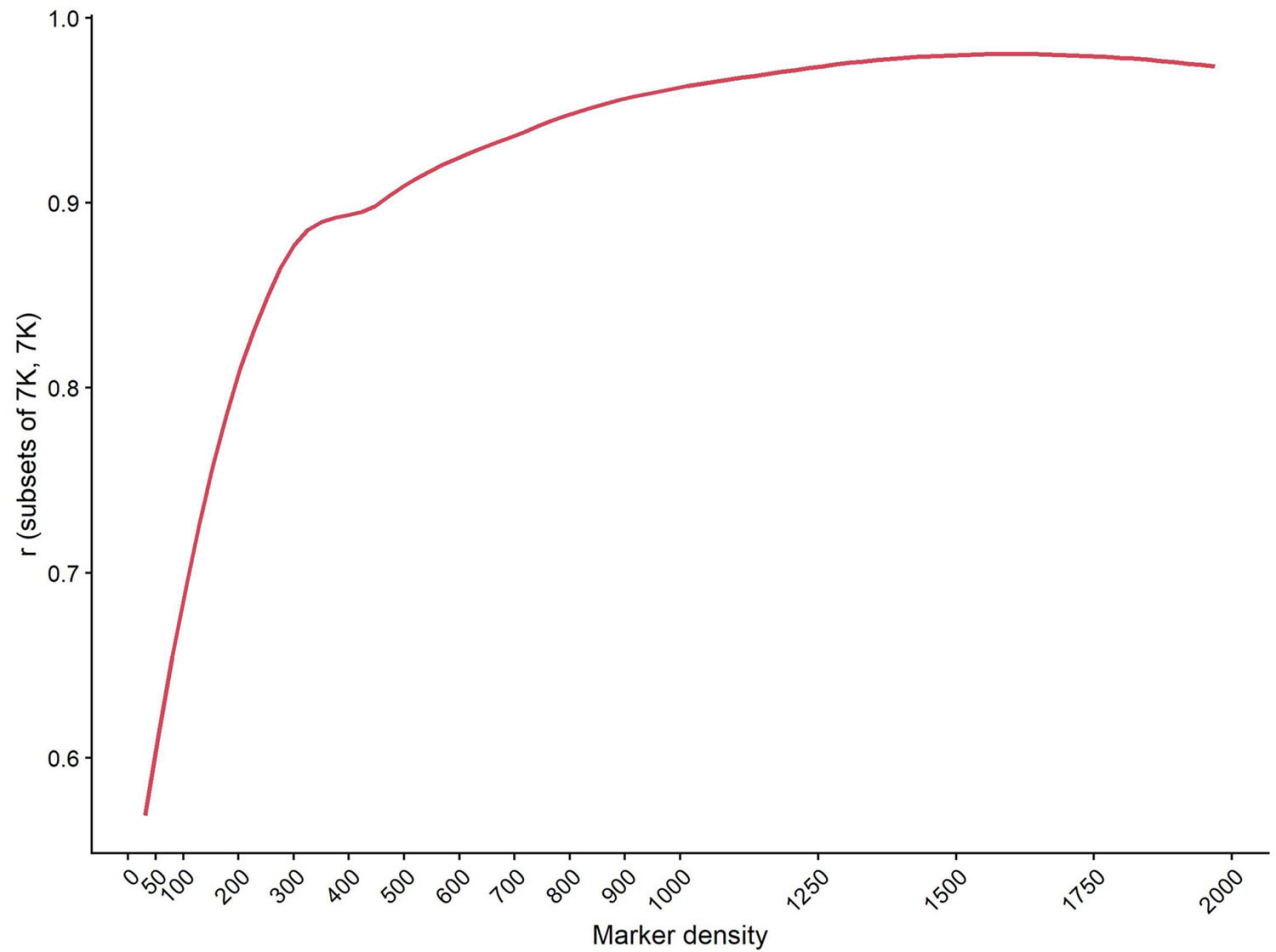
Correlation between additive relationship coefficients calculated with subsets of the C7AIR rice single‐nucleotide polymorphism array (7K) and the full 7K array

**FIGURE 3:**
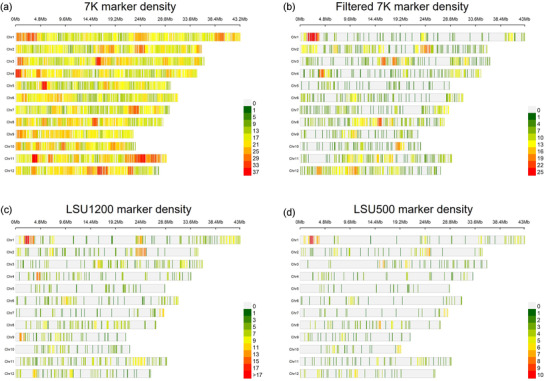
Marker density across different marker sets. (a) C7AIR rice single‐nucleotide polymorphism (SNP) array (7K) marker density; (b) 7K filtered with minor allele frequency >0.1 and missing data <20% marker density; (c) LSU1200 marker density; (d) LSU500 marker density. Colors refer to number of single‐nucleotide polymorphisms within 1‐Mb window size

To identify a subset of the 7K markers for routine genotyping, the additive relationship coefficients of the RGS lines calculated using the 7K polymorphic markers were correlated with the coefficients calculated using smaller marker densities (Figure 2). No increase was observed beyond 1,200 markers. A marginal decrease occurred at 300 markers and a significant reduction below 300 markers. To select the most informative SNPs from the 7K array, markers with >20% missing data and MAF < 0.1 were removed, resulting in 1,935 markers (Figure 3). These markers were then used to characterize haplotype blocks and select the minimum number of SNPs needed to tag the different regions. The total number of optimal tag SNPs was 1,174. In addition, 28 trait‐specific and 16 genome‐wide KASP markers used routinely in the breeding program were added, resulting in a total of 1,218 markers, hereafter referred to as ‘LSU1200’. The average MAF of these markers across the RGS lines was 0.18, while the average distance between markers was 291 kbp. These markers were tested across all target germplasm using the AmpSeq genotyping platform of the genotyping service provider Agriplex Genomics (https://agriplexgenomics.com/). A set of 4,230 experimental lines was genotyped with the LSU1200, consisting of 2,067 lines from PYTs, 1,779 lines from six unique biparental mapping populations, and 384 lines from a U.S. rice breeding germplasm, the DP. Two hundred and five markers had >20% missing data and were excluded, while 209 samples failed. Thus, a total of 1,013 SNPs (17% failure rate) remained for subsequent analysis. When comparing the calculated relationship coefficients, the LSU1200 had a correlation of 0.96 with the 7K array and 0.63 with the pedigree information, demonstrating its ability to reliably capture the overall genetic variation (Supplemental Table S3).

#### 3.1.3 LSU500 marker set design

Upon identifying the 1,013 SNPs that converted well to the AmpSeq platform, analysis was conducted to determine whether a more streamlined marker set might be useful for implementation of GS. The primary goal was to reduce the size of the marker set to minimize costs for routine genotyping without significantly decreasing the ability to capture the genetic variation of the target germplasm. Correlation between the relationship coefficients calculated with different marker densities (Figure 2) showed that marker densities above 400 markers were still highly correlated to the 7K array (*r* > 0.85).

To explore the potential of minimizing the size of the LSU1200 marker set, the 384 lines of the DP were used to compare the ability of smaller marker densities to estimate the genetic relationship between lines (Supplemental Figure S2). The correlation of different marker subsets of the LSU1200 to the full LSU1200 set was above 0.95 with marker densities above 200, 0.98 with 500 markers, and the correlation did not increase with marker densities above 600. Based on this observation, a marker set of 550 SNPs, the LSU500, was designed by removing markers with lower call rates and prioritizing SNPs with higher MAF among closely located markers (Figure 3; Supplemental Table S2). The average MAF across the RGS lines was 0.22, with average distance of 663.1 kb. The LD calculation within the biparental populations and all the PYTs breeding lines separately showed that LD in the breeding program is high (∼10 Mb) (Supplemental Figure S3); and therefore, a well‐chosen set of ∼300 markers can tag the chromosomal segments segregating within the breeding population. The LD within the biparental populations was higher than the PYTs. The correlation between the additive relationship coefficients calculated with the LSU500 and the 7K array was 0.93 and 0.99 with LSU1200. The correlation with the relationship coefficients calculated with pedigree information was 0.63, consistent with what was observed with the LSU1200 (Supplemental Table S3). To compare the efficiency of the LSU500 and LSU1200 marker sets for GS, a CV was performed to measure the predictive ability of the two sets across six traits within two Clearfield long‐grain PYTs conducted in 2018 and 2019, consisting of 750 different entries in each trial. The results of the CV were, on average, nearly identical between the two marker sets for each trait and trial, with mean values ranging from 0.19 for chalk to 0.69 for plant height (Supplemental Figure S4).

### 3.2 LSU500 for genomic selection

#### 3.2.1 Predictive ability across preliminary yield trials

**FIGURE 4:**
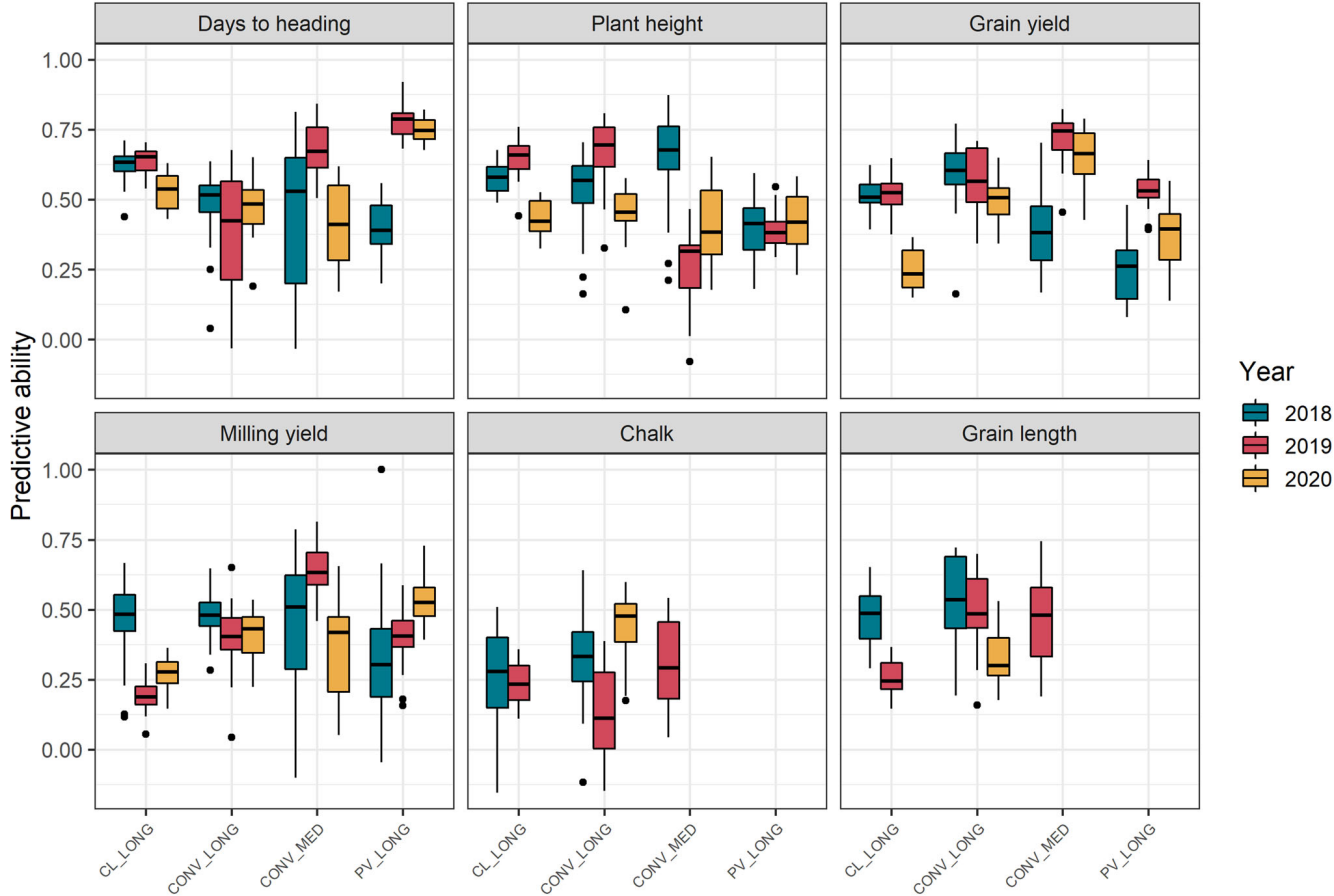
Cross‐validation conducted within each breeding market class segment preliminary yield trial. Boxplots show the predictive ability for each trait and trial

Once the LSU500 set was determined to be suitable for routine GS, the remaining 6,406 experimental lines of the breeding program pipeline from the three most recent years (2018–2020) were genotyped to serve as a training population for future predictions. A CV was conducted on 4,078 unique breeding lines (8,172 observations) tested in four different PYTs grown at the HRCRRS in 2018, 2019, and 2020 to evaluate GS within the different segments of the breeding program, Clearfield long‐grain (CL_LONG), conventional long‐grain (CONV_LONG), conventional medium‐grain (CONV_MED), and Provisia long‐grain (PV_LONG) (Figure 4). The predictive ability was influenced by the narrow‐sense heritability (*h^2^*) (Table 1) of each trait–trial combination and by the dimension of the training set used in the CV. Lower narrow‐sense heritability traits, such as chalk, produced lower predictive ability, and smaller trials, like conventional medium‐grain, where the training population was small, produced lower predictive abilities despite average estimates of heritability. On average, the highest predictive ability was observed for days to heading (0.55); grain yield and plant height were similar (0.49), while grain length (0.43) and milling yield (0.41) had slightly lower values. The lowest values were observed for chalk (0.29). Overall, this marker set produced good predictive abilities across different trials; however, large differences can still be observed within different trials (Figure 4).

**TABLE 1:** Average broad‐sense heritability (*H* ^2^) and narrow‐sense heritability (*h* ^2^) calculated across four market classes preliminary yield trials grown from 2018 to 2020

	H^2^	h^2^
Grain yield	.59	.53
Days to heading	.79	.75
Plant height	.66	.59
Milling yield	–	.36
Grain length	–	.44
Chalk	–	.18

*Note*. *H* ^2^ is not reported for traits based on single measurements.

#### 3.2.2 Genomic selection and phenotypic selection

**FIGURE 5:**
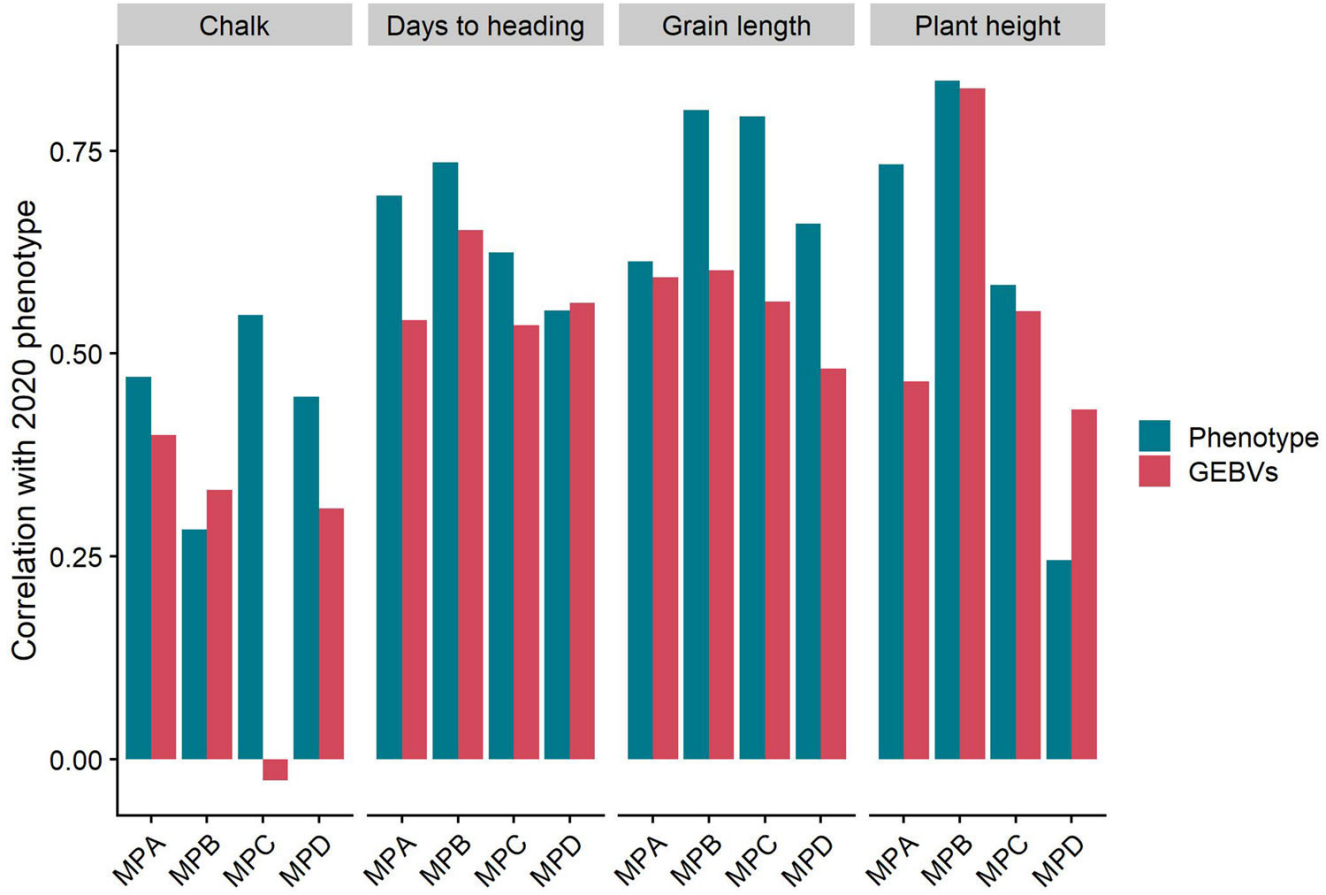
Correlation of 2019 phenotype and 2019 genomic estimated breeding values with the observed 2020 phenotype within four biparental populations (MPA, MPB, MPC, MPD)

The predictive ability from CV across PYTs (Figure 4; Supplemental Figure S4) was calculated by correlating the GEBVs with the phenotype of a single year‐one location PYT, thereby assuming the single‐year phenotype as the true breeding value of a line. However, a more relevant estimate of the GS potential for applied breeding applications should be calculated comparing both the ability of GEBVs and phenotype to predict future performances of a line. To make this comparison, phenotype data collected in 2019 from MPA, MPB, MPC, and MPD biparental populations was used to calculate GEBVs and compare them and the 2019 phenotype data to the observed 2020 phenotype (Figure 5). Within each population, the LSU500 marker set contained an average of 265 polymorphic markers with MAF > 0.05, ranging from 243 to 297. Across the four populations, the average GS predictive ability compared with phenotype for plant height was 0.57 and 0.60, respectively; for days to heading, it was 0.57 and 0.65, for grain length it was 0.56 and 0.72, and for chalk it was 0.26 and 0.44, respectively However, both GS predictive ability and phenotype differed across traits and across populations (Figure 5). Across the 16 different trait–population comparisons, the reduction of accuracy of the GEBVs vs. the phenotype was 20% or less in nine cases. In three cases, the GEBVs were more accurate than the phenotype.

Within the MPC population, very low prediction accuracies were observed for chalk, while the other traits within the MPC had similar accuracies as observed in the other populations. This observation may be explained by sparse marker coverage within the MPC population in the genomic regions underlying the variation of the trait in the population.

### 3.3 Moving forward: reducing marker density

#### 3.3.1 Marker density across experimental trials

**FIGURE 6:**
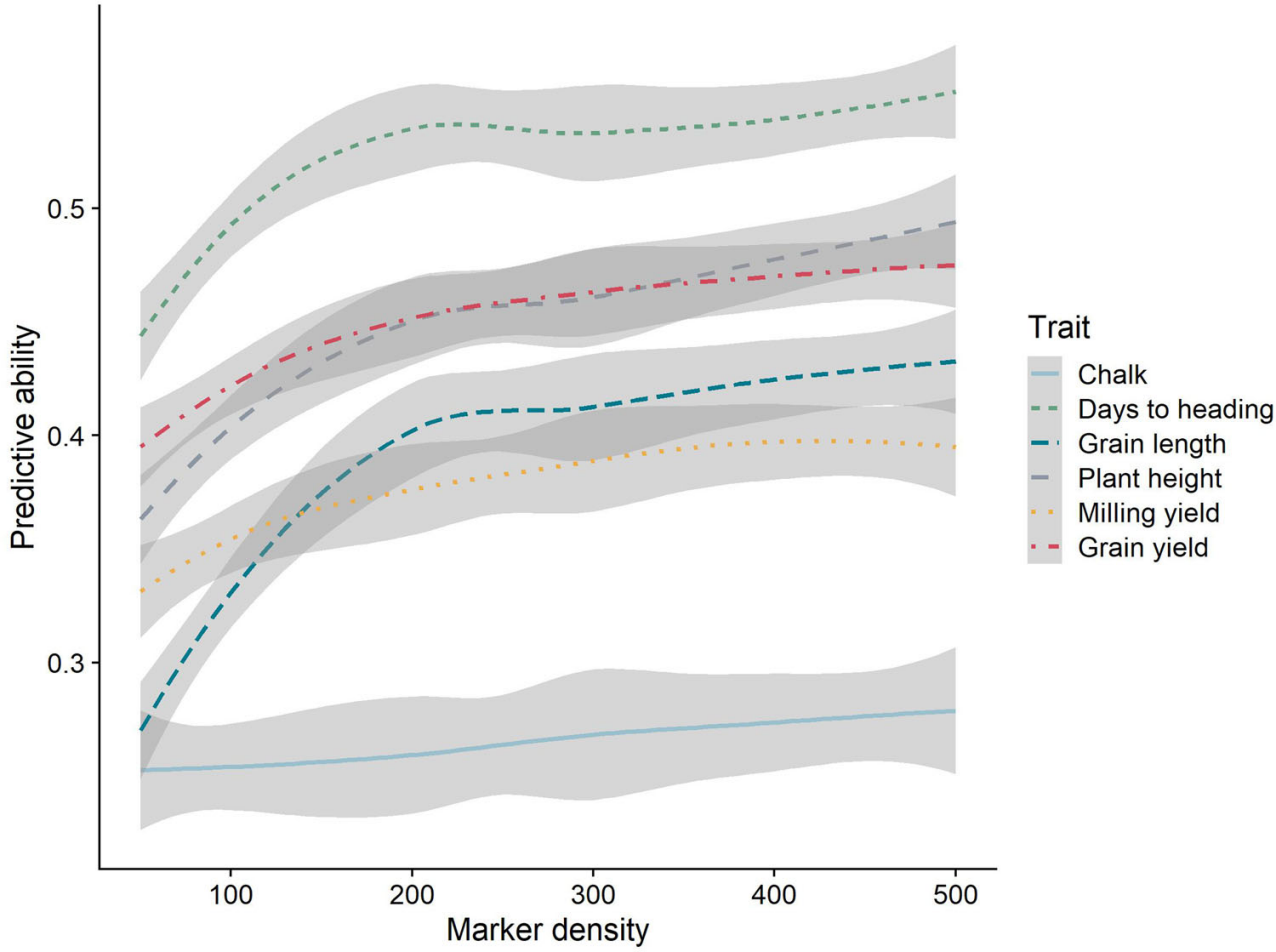
Average cross‐validation predictive ability across different marker densities. Cross‐validation was conducted within each breeding market class segment using the 2018, 2019, and 2020 preliminary yield trials. Lines show average values across all trials for each marker density. The shaded areas show 90% confidence interval around the average value

The ability of the LSU500 marker set to capture the genetic variation of the target breeding germplasm is nearly identical to the LSU1200 marker set (Supplemental Table S3). However, it was observed that marker subsets below 500 were effective at capturing the relationships among the germplasm as well (Figure 2). If it is possible to further reduce the marker set in the future without impacting the predictive ability, this would be desired from a cost standpoint. To investigate the potential to further reduce the size of the marker set, the average predictive ability of CV within the PYTs was compared across different marker densities by removing adjacent markers at increasing physical distances (Figure 6). Overall, no differences were recorded with marker densities as low as 200, except for plant height. Different trends across different marker densities were observed across the different traits. Overall, higher predictive ability traits with the full set had higher predictive ability with smaller densities except for plant height and grain length, which were more affected by lower marker densities than other traits.

#### 3.3.2 Marker density within biparental populations

To quantify the impact of marker density reduction within biparental families, the correlation of GEBVs with the phenotype of the following year was compared across different populations and marker densities (Supplemental Figure S5). On average, the predictive ability for days to heading did not show major variation across marker densities of 500, 400, 300, and 200 with values ranging from 0.57 to 0.58. It decreased to 0.37 with 100 markers and to 0.25 with 50 markers. Grain length predictive ability (0.56) decreased gradually with densities below 400 markers reaching 0.42 predictive ability with 50 markers. Plant height predictive ability (0.57) decreased gradually with smaller marker densities reaching 0.27 with 50 markers. Chalk predictive ability increased with lower marker densities from 0.25 up to 0.32 with 100 markers and then decreased to 0.22 with 50 markers.

## 4 DISCUSSION

### 4.1 LSU500 for genomic selection implementation

The objective of this work was to develop and validate a mid‐density marker set for routine genotyping of southern U.S. rice breeding lines and to determine its usefulness for GS. The LSU500 marker set is designed for multiplex AmpSeq and is available for outsourcing at the genotyping service provider Agriplex Genomics (https://agriplexgenomics.com/). This marker set addresses the need for a low‐cost, efficient, and germplasm‐tailored platform ideal for GS in southern U.S. rice. A practical example of the initial steps of defining a genotyping method, developing a preliminary marker set, and testing the marker set suitability for GS was presented. This process can be difficult since it requires multiple different skill sets that are not always present in a traditional breeding program (Santantonio et al., 2020). The output of this work clearly demonstrates the utility of the LSU500 marker set for GS applications and highlights the utility of GS in U.S. rice breeding germplasm.

The design of the set was done by including SNPs from the 7K array (Morales et al., 2020) that capture the genetic variation of global rice germplasm and some key trait markers. The fact that the 7K array has utility across global germplasm suggests that it is not likely ideal for a specific breeding program germplasm. This was highlighted by the low MAF (<0.05) of over 60% of the 7K SNPs across our target germplasm. Similarly, adding additional markers with low MAF did not increase prediction accuracy as shown in Figure 1 for 7,098 and 4,000 markers. The high LD in the breeding population vs. the LD in global rice facilitated the reduction of the marker density found in the 7K array without losing predictive ability. The SNPs were selected based on their ability to tag individual haplotypes of the breeding population; with this approach, the predictive ability of this set was similar to the full 7K array in a CV experiment. In addition, the ability of the LSU500 to capture the relationships between breeding lines was comparable with the 7K array.

One hurdle of implementing GS in applied breeding programs is the cost of genotyping. To minimize genotyping cost, it is critical to limit the number of markers by optimizing the marker set for the target germplasm. The LSU500 marker set was selected specifically for southern U.S. rice germplasm and all markers are all highly informative within the target germplasm.

The cost of genotyping with the LSU500 is less than the cost of testing an experimental line in a single‐location PYT. The cost of outsourcing the genotyping with the 7K array is between $35 and $40, genotyping‐by‐sequencing (GBS) is approximately $25, the LSU1200 is $10, and our current cost per sample with the LSU500 is $4.80 through Agriplex Genomics. The cost of producing the seed at the winter nursery to be used in the PYT is over $10 per entry. This is double the cost of genotyping with the LSU500 and does not include the cost of conducting the field trial and collecting all the phenotype data. Agriplex Genomics offers a 4–6 wk genotyping turnaround time, which is sufficient for our breeding activities and calendar. A significant consideration to implementing GS for our program centers around the logistics of routine activities and ensuring that key breeding processes are not delayed. Geographic location of the genotyping service provider within the United States was also an important consideration to reduce shipping time, phytosanitary constraints, and paperwork.

Similarly, the 1k‐RiCA marker set is a previously developed rice marker set also available through Agriplex; however, it is more informative within *indica* rice germplasm (Arbelaez et al., 2019).

### 4.2 LSU500 and genomic selection

The LSU500 marker set enables GS across different traits and groups of germplasm at a level of accuracy that is sufficient for implementation in a breeding program. When testing GS with CV across different trials and environments, the predictive ability varied significantly according to the heritability of the trait–trial combination and the size of the training population used in the CV. It is important to note that the heritability of a trait in a specific trial or environment is affected by many aspects, such as the accuracy of the phenotyping method, the trait genetic architecture, the phenotypic variation of the environment, and the presence of large differences between the genotypes tested (Covarrubias‐Pazaran, 2019).

Population structure is another important factor to account for in GS experiments. Predictive ability estimates can be biased relative to accuracy obtained in practice if family size and structure differ between the CV dataset and real‐life prediction scenarios (Werner et al., 2020). For example, in the early stages of a breeding program, many full‐sib and half‐sib individuals are tested together. This contrasts with later stages where there are many different families represented, each of small size. Thus, in earlier stages, prediction is largely within families, and at later stages, prediction is across families. Both scenarios were considered by testing prediction within biparental populations (early stage) and PYTs (later stage).

Four biparental populations were phenotyped for key traits across 2 yr, providing the opportunity to compare the correlation of the GEBVs vs. correlations between phenotypes measured in different years. This is a useful measure of the predictive ability of GS and provides additional context when compared with using CV alone. In the CV experiments, the prediction accuracy was calculated by comparing the GEBVs with the phenotypes observed in the CV experiment, which assumes that the observed phenotypes are the true breeding value of the line. However, for the traits explored in these populations across 2 yr, the correlation of the phenotypes was on average 0.60, ranging from 0.25 to 0.84 across Year 1 and Year 2. When compared with the predictive ability of GS using Year 1 as the training set, the correlation of the GEBVs to the Year 2 phenotypes was, on average, 0.49, ranging from −0.02 to 0.83. These results confirmed that GS based on the LSU500 genotypes produced good predictive ability across full‐sibs and different biparental populations. However, the greatest gains from GS are obtained by predicting the performance of experimental lines that have never been tested. Therefore, further analysis should be conducted using training populations of related historical lines for predictions to measure the GS ability to predict the future performance.

### 4.3 Beyond the LSU500

The objective of the LSU500 is to provide a robust marker set for implementation of GS in southern U.S. rice breeding programs. However, the LSU500 is not intended to be the final or fixed marker set moving forward. This initial marker set will enable U.S. rice breeding programs to rapidly and economically explore the potential of GS in their materials. The design and implementation of the LSU500 is following a continuous integration strategy (Supplemental Figure S6), whereby GS is initially introduced into a breeding program while technical and strategic aspects of GS implementation are evaluated, optimized, and integrated into the breeding pipeline on the go. This strategy allows resources to be invested progressively and reveals possible implementation problems as early as possible. We will continue to improve upon the marker set as more data and information are obtained. The ability to easily update the marker set is another benefit of the Agriplex AmpSeq platform over existing fixed assays. Future iterations of the GS marker set will aim to address some physical gaps throughout the genome and regions of the genome in which the existing markers may not tag independent chromosomal segments. The inability of the LSU500 marker set to predict chalk in the biparental population MPC is an example where increasing marker coverage may improve the predictability of the trait. One reason for the presence of regions of low marker coverage can be traced to the original source of SNPs, the 7K array; when selecting markers from a specific array, gaps within the array are inevitably present in the selected subset. To address this issue, we generated whole‐genome sequence for the 384 lines of the DP selected to represent the genetic diversity of southern U.S. rice germplasm including important founders. These data will be used to select new informative markers to augment the LSU500 set.

The LSU500 represents a short‐term solution for starting GS in the breeding program. The next stage of our marker strategy will focus on imputation. Imputing progeny from low marker density to high‐density data obtained for parents can greatly increase prediction accuracy and reduce genotyping costs (Jacobson et al., 2015). Results from varying marker densities across PYTs and biparental populations suggest that the number of markers could be further reduced if an imputation strategy is adopted. Because of high LD in biparental populations, imputation of marker information from a lower density (100–500 markers) to higher density (∼10,000 markers) datasets obtained on parents can be achieved with high accuracy (Gonen et al., 2018). In a plant breeding program, a new breeding cohort is initiated every year by crossing elite parents. The group of parents is generally small, ∼100, and some will be repeated across years. As a result, it is feasible to sequence new parents every year so that each breeding line can be imputed using its direct parents (Gorjanc et al., 2017). In addition, the cultivars considered as the founders of the breeding program germplasm have whole‐genome sequence data that can be used to impute to even higher densities using tools like the practical haplotype graph (Jensen et al., 2020).

Following this strategy, we used the DArTSeq genotyping technology through the genotyping service provider Diversity Array Technology in Australia (https://www.diversityarrays.com/) to genotype 188 lines that have been used in our crossing blocks since 2017. This genotyping method provides higher DNA marker information (∼10,000 SNPs) for a reduced cost per sample, compared with whole genome sequencing, and will be used for imputation purposes and to monitor program diversity. Information from the whole‐genome sequence data of the 384 DP founders will be used as the basis for imputation to increase the density of genotypic information on the lines from the crossing blocks.

Moving forward, when integrating GS into the routine breeding pipeline, it is important that the markers remain informative as the breeding program evolves. When recycling advanced lines and bringing in new material for crossing, allele frequencies are altered, LD patterns are broken, and new alleles are introduced. For these reasons, the marker set for routine genotyping will need to be updated on a regular basis.

## 5 CONCLUSION

In this paper, we present our strategy for developing a routine genotyping assay, discuss the results of our efforts to date, and present our vision for how this assay will evolve in the future. Genomic selection promises to bring great advantages to a breeding program, including improving the accuracy of selection and increasing the number of tested experimental lines, both while reducing the cost and duration of operations. At the same time, implementation of this breeding approach requires significant changes to the existing breeding program's structure and practice. Multiple logistical barriers need to be addressed for the adoption of GS to be successful. A custom‐tailored marker set for AmpSeq that is informative for the breeding program's germplasm and can be outsourced to a third‐party genotyping provider is an essential ingredient for a rapid and cost‐effective initial implementation of GS in the breeding program. The LSU500 is a reliable and efficient marker set with demonstrated utility for routine genotyping of rice in a southern U.S. rice breeding program. It is currently available at a cost‐effective price from the genotyping service provider, and, in the future, it will undergo improvements to reduce the cost and increase the accuracy of GS. Moreover, because of the complex nature of GS implementation and the peculiarity of every breeding program, the genotyping method and GS implementation strategy will require further evaluation and customization to meet each program's specific needs.

## AUTHOR CONTRIBUTIONS

Tommaso Cerioli: Conceptualization; Data curation; Formal analysis; Investigation; Writing – original draft. Christopher O. Hernandez: Conceptualization; Data curation; Formal analysis; Visualization; Writing – original draft; Writing – review & editing. Brijesh Angira: Data curation; Investigation; Writing – review & editing. Susan R. McCouch: Conceptualization; Investigation; Methodology; Writing – review & editing. Kelly R. Robbins: Conceptualization; Investigation; Methodology; Project administration; Supervision; Writing – review & editing. Adam N. Famoso: Conceptualization; Funding acquisition; Investigation; Methodology; Project administration; Supervision; Writing – review & editing.

## CONFLICT OF INTEREST

The authors declare no conflict of interest.

## Supporting information

Supplemental Table S1 Summary of material genotyped and tested in this study
Supplemental Table S2 List of markers included in the LSU1200 alone, and LSU1200 and LSU500. See attached .xlsx file
Supplemental Table S3 Correlation between additive relationship matrices calculated with pedigree information, C7AIR rice SNP array (7K), LSU1200 marker set, and LSU500 marker set of 342 lines of the representative germplasm set.
Supplemental Code Python code used to select the minimum number of SNP markers needed to uniquely identify each haplotype block See attached word file.

Supplementary Information

Supplementary Information

Supplementary Information

Supplementary Information

Supplementary Information

Supplementary Information

Supplementary Information

Supplementary Information
