# Supplementary material for: Development and validation of an optimized marker set for genomic selection in southern U.S. rice breeding programs
Source: Plant Genome. 2022 May 25;15(3):e20219. doi: 10.1002/tpg2.20219 (PMC12806970; doi:10.1002/tpg2.20219)
Supplement: Supplementary file 1 [file TPG2-15-e20219-s002.docx]

#!/usr/bin/python3

import sys

import pandas as pd

import numpy as np

def main():

hapBlocks, genoMat, minPIC = sys.argv[1:] # Unpack arguments

blocks = read_hap(hapBlocks) # Read in haploblocks

geno = read_genoMat(genoMat) # Read in genotype matrix

outFile = hapBlocks.split(".blocks.det")[0] + ".tags"

for block in blocks: # Get PIC info for blocks

calcPIC(block, geno)

for block in blocks: # Pick tagSNPs for each block

tagger(block)

print(blocks[4].get_alleles())

print(blocks[4].get_tags())

print(blocks[4].get_genotypes())

with open(outFile, "w") as fh: # Write out tag snp info

fh.write("chrom\tstart\tend\tlen\tsnpN\ttagN\ttagIDs\tPIC\talleleN\n")

for block in blocks:

fh.write(block.get_info() + "\n")

def read_hap(hapBlocks):

"""Reads in PLINK .blocks.det file"""

blocks = []

with open(hapBlocks) as fh:

_ = fh.readline()

for ID, line in enumerate(fh):

tmp = line.strip().split()

tmp = [x.strip() for x in tmp] # Extra cleanup

tmp[-1] = [x.strip() for x in tmp[-1].split("|")]

blocks.append(Block(ID=ID, chrom=int(tmp[0]), start=int(tmp[1]),

end=int(tmp[2]), length=float(tmp[3]),

snpN=int(tmp[4]), snps=tmp[-1]))

return(blocks)

def read_genoMat(genoMat):

"""Read in genotype matrix"""

geno = pd.read_table(genoMat)

return(geno)

def removeHet(genotype):

"""Removes heterozyougs genotypes from blocks"""

hets = set(["R", "Y", "S", "W", "K", "M"])

if "N" not in genotype:

if len(hets.intersection(set(genotype))) == 0:

return(True)

else:

return(False)

else:

return(False)

def pairwiseMult(array):

"""Calculates 2 * pairwise combinations of a 1D array"""

res = []

for i in range(len(array)):

for j in range(len(array)):

if i != j:

res.append(array[i] * array[j])

return(res)

def toMat(genotypes, snps):

final = []

for i in genotypes:

tmp = []

for s in i:

tmp.append(s)

final.append(tmp)

return(pd.DataFrame(final, columns=snps))

def calcPIC(block, geno):

"""Gets block allele, PIC score, and genotype info"""

snps = block.snps

genotypes = map(lambda x : "".join(x), geno[snps].values)

genotypes_filtered = list(filter(removeHet, genotypes))

alleles, count = np.unique(genotypes_filtered, return_counts=True)

freq = count/sum(count)

freqSq = freq * freq

PIC = 1 - sum(freqSq) - 2 * sum(pairwiseMult(freqSq))

block.add_PIC(PIC)

block.add_alleles(alleles)

block.add_genotypes(toMat(genotypes_filtered, snps))

def tagger(block):

"""Greedily picks the minimium set of SNPs

for discrimating haploblock alleles"""

alleleN= len(block.alleles)

geno = block.genotypes

classes = 0

snps = block.snps

tags = []

while classes < alleleN:

best = 0

bestSNP = ""

for s in snps:

sub = [s] + tags

genotypes = map(lambda x : "".join(x), geno[sub].values)

alleles = np.unique(list(genotypes))

if len(alleles) > best:

best = len(alleles)

bestSNP = s

classes = best

tags.append(bestSNP)

snps.remove(bestSNP)

block.add_tags(tags)

class Block(object):

"""Class for holding haplotype block information"""

def __init__(self, ID, chrom, start, end, length, snpN, snps):

self.ID = ID

self.chrom = chrom

self.start = start

self.end = end

self.length = length

self.snpN = snpN

self.snps = snps

self.tagSnps = None

self.PIC = None

self.alleles = None

self.genotypes = None

def add_tags(self, tags):

self.tagSnps = tags

def add_PIC(self, PIC):

self.PIC = PIC

def add_alleles(self, alleles):

self.alleles = alleles

def add_genotypes(self, genotypes):

self.genotypes = genotypes

def get_tags(self):

return(self.tagSnps)

def get_PIC(self):

return(self.PIC)

def get_alleles(self):

return(self.alleles)

def get_genotypes(self):

return(self.genotypes)

def get_info(self):

info = "%i\t%i\t%i\t%f\t%i\t%i\t%s\t%f\t%i" % (self.chrom, self.start, self.end, self.length,

self.snpN, len(self.tagSnps),

"|".join(self.tagSnps), self.PIC,

len(self.alleles))

return(info)

if __name__ == "__main__":

main()
