# Supplementary material for: Development and validation of an optimized marker set for genomic selection in southern U.S. rice breeding programs
Source: Plant Genome. 2022 May 25;15(3):e20219. doi: 10.1002/tpg2.20219 (PMC12806970; doi:10.1002/tpg2.20219)
Supplement: Supplementary file 2 [file TPG2-15-e20219-s003.docx]

**Supplemental Table S1** Summary of material genotyped and tested in this study

| **Genetic Material** | **Description** | **Marker Set** | **Phenotype Measured** |
| --- | --- | --- | --- |
| Representative Germplasm Set (RGS) | 66 modern and historical varieties + 276 experimental lines from 2017 Clearfield Long Grain Preliminary Yield Trial | 7K | Days to heading, plant height, milling yield, grain yield |
| Diversity Panel (DP) | 384 lines that represent Southern rice breeding germplasm and accessions from different regions of the world | LSU1200 |  |
| Bi-parental populations | MPA, MPB, MPC, MPD - Four bi-parental populations representing elite US Southern rice germplasm - 1,200 experimental lines | LSU1200 | Days to heading, plant height, chalk, grain length |
| Preliminary Yield Trials  (PYTs) | Clearfield Long Grain PYTs from 2018 and 2019 (CL_LONG_18 and CL_LONG_19) - 1,500 experimental lines | LSU1200 | Days to heading, plant height, chalk, grain length, milling yield, grain yield |
|  | All PYTs grown from 2018 to 2020 for Clearfield Long Grain, Conventional Long Grain, Conventional Medium Grain, Provisia Long Grain (CL_LONG, CONV_LONG, CONV_MED, PV_LONG) - 6,406 experimental lines | LSU500 | Days to heading, plant height, chalk, grain length, milling yield, grain yield |

**Supplemental Table S2** List of markers included in the LSU1200 alone, and LSU1200 and LSU500. **See attached .xlsx file**

**Supplemental Table S3** Correlation between additive relationship matrices calculated with pedigree information, C7AIR rice SNP array (7K), LSU1200 marker set, and LSU500 marker set of 342 lines of the representative germplasm set.

|  | **Pedigree** | **7K** | **LSU1200** | **LSU500** |
| --- | --- | --- | --- | --- |
| **Pedigree** | 1 |  |  |  |
| **7K** | 0.67 | 1 |  |  |
| **LSU1200** | 0.63 | 0.96 | 1 |  |
| **LSU500** | 0.63 | 0.93 | 0.99 | 1 |

**Supplemental Code** Python code used to select the minimum number of SNP markers needed to uniquely identify each haplotype block **See attached word file**
